# Citrate of Soda in Diabetes

**Published:** 1866-10

**Authors:** 


Citrate of Soda in Diabetes.—M. Guyot-Danecy, basing his practice upon the theory that diabetes arises from imperfect combustion of the glucose of the blood, proposes to employ citrate of soda in order to supply the alkaline carbonate which is necessary to the progressive chemical change of the glucose. He substitutes the citrate for the carbonate, because, he says, it does not affect the function of digestion. He administers the salt in doses of from four to eight grammes. His analysis, he alleges, demonstrate that sugar disappears from the urine after the administration of the citrate. Citrate of soda may be mixed with food instead of salt, and with it the use of ordinary bread and starchy matters ceases to be objectionable.—*Lancet.*

